# Physical health in subjects with Schizophrenia: where are we?

**DOI:** 10.1192/j.eurpsy.2025.184

**Published:** 2025-08-26

**Authors:** A. Mucci

**Affiliations:** Mental and Physical Health and Preventive Medicine, University of Campania L. Vanvitelli, Naples, Italy

## Abstract

**Abstract:**

People living with schizophrenia (PLWS) face one of the most significant health equality gaps in Europe. Their life expectancy is 15–20 years shorter than that of the general population, mainly because they are affected at an earlier age by preventable physical illnesses, but encounter barriers in accessing adequate care. PLWS did not benefit from prevention campaigns for cardiovascular, oncologic, or metabolic risk. Antipsychotics might add to the cardiometabolic risk and represent a further reason for monitoring and treating emergent conditions. Notwithstanding international guidance papers or national guidelines, PLWS do not receive adequate screening and treatment.

This presentation will summarize national and international efforts to reduce this health equality gap, illustrating the minimum screening procedures and several interventions that can be integrated into schizophrenia treatment to improve health outcomes of PLWS.

**Disclosure of Interest:**

A. Mucci Consultant of: Angelini, Gedeon. Richter Bulgaria, Janssen Pharmaceuticals, Lundbeck, Otsuka Pharmaceutical, Pfizer, Pierre Fabre, Rovi. Pharma and Boehringer Ingelheim

